# Supplementary figures and images for: Norovirus evolution in immunodeficient mice reveals potentiated pathogenicity via a single nucleotide change in the viral capsid
Source: PLoS Pathog. 2021 Mar 11;17(3):e1009402. doi: 10.1371/journal.ppat.1009402 (PMC7987144; doi:10.1371/journal.ppat.1009402)

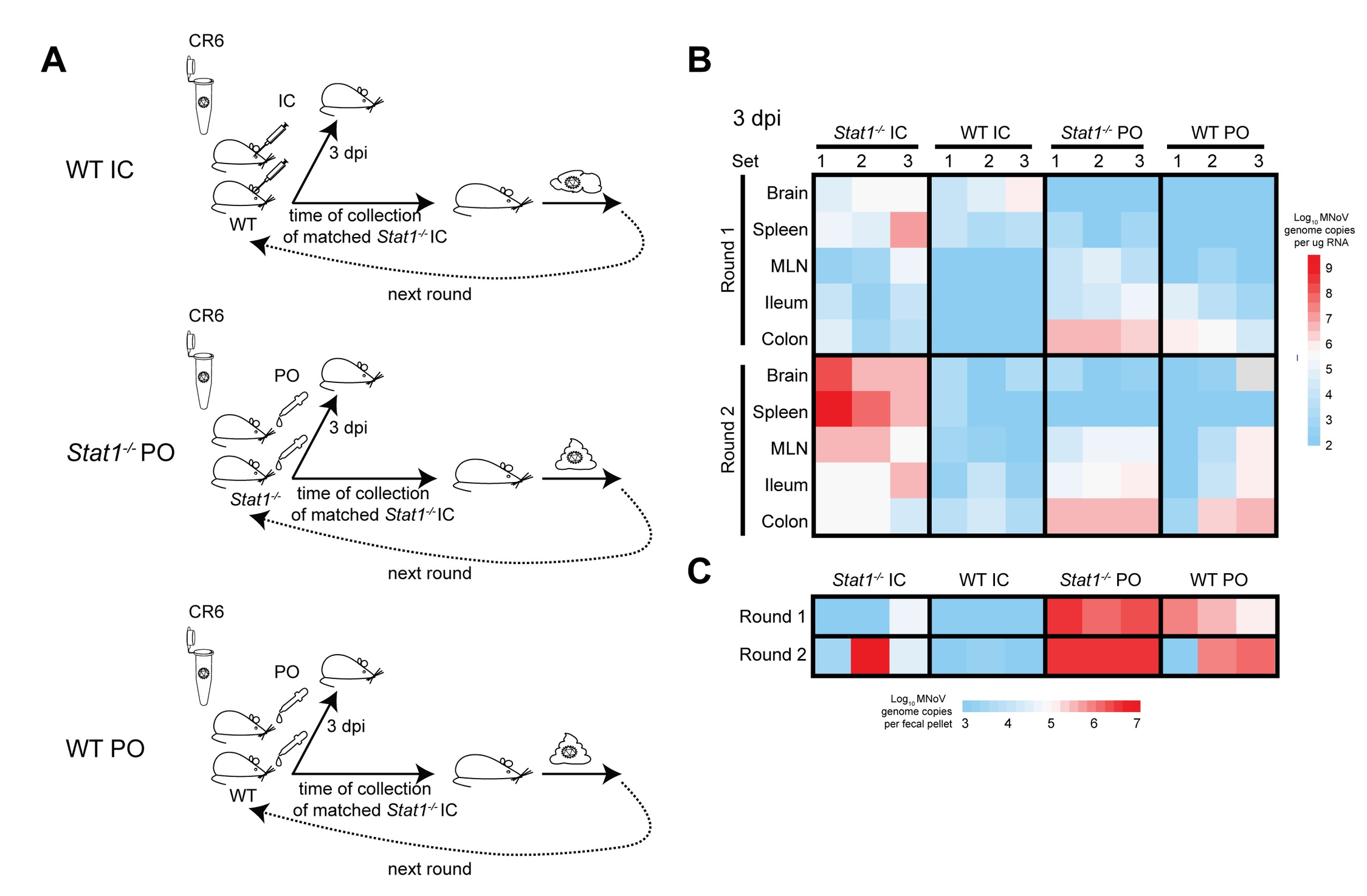

Supplement: S1 Fig — (A) Schematics for control groups including intracranial (IC) passaging protocol in WT mice as well as per oral (PO) passaging protocols in WT and Stat1-/- mice, all initiated with 106 PFU CR6 in three parallel “sets” of mice. Sacrifice of mice in these control groups was matched with spontaneous time of death of Stat1-/- IC experimental groups. For PO passaging, fecal material collected from mice at time of sacrifice was administered orally to the next round of mice. (B,C) Heatmap depicting murine norovirus levels detected by quantitative RT-PCR (qPCR) in designated tissues (B) or stool (C) at 3 dpi, with values ranging from log10 1.95 to 9.50 (limit of detection at 2.0) for tissues and log10 2.95 to 6.95 (limit of detection at 3.0) for stool. Each square indicates a single mouse tissue from 24 total mice analyzed. (TIF) [file ppat.1009402.s001.tif]

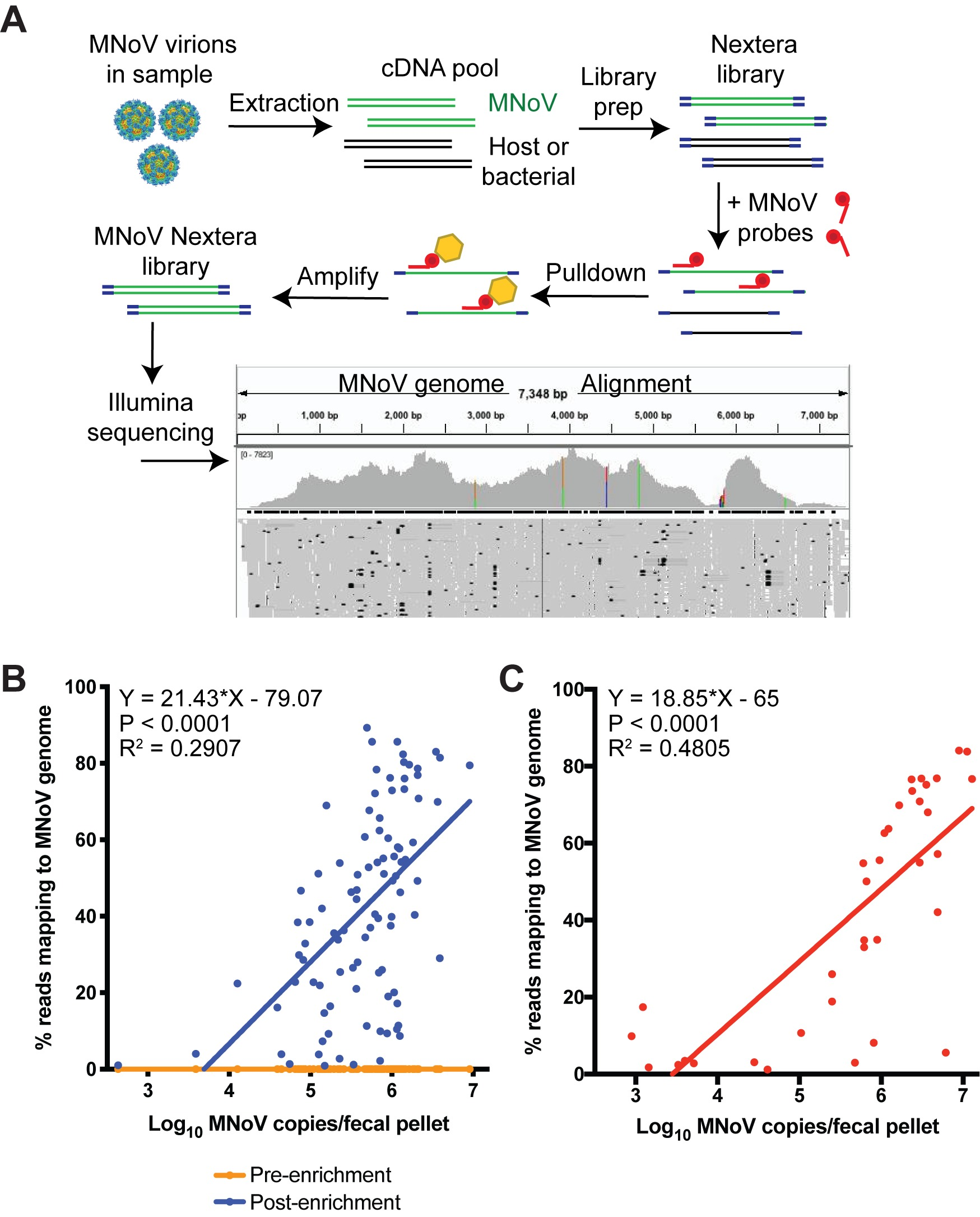

Supplement: S2 Fig — (A) Schematic of enrichment protocol, in which cDNA from stool RNA undergoes Nextera tagmentation, followed by hybridization capture using biotinylated MNoV-specific probes and streptavidin beads (yellow hexagons) for pulldown prior to Illumina sequencing. (B) Stool samples collected from various mice infected PO with CR6 underwent this protocol, then pre- and post-enrichment samples were sequenced to assess efficacy of enrichment. (C) For stool samples from this study (Figs 1D and S1C) that were deep-sequenced after enrichment, the percentage of reads mapping to MNoV were compared to viral levels detected in samples by qPCR. Linear regression analysis for post-enrichment samples (B) and samples for this study (C) was performed using GraphPad Prism 7 software, with the P-value reporting if the slope is significantly non-zero. (TIF) [file ppat.1009402.s002.tif]

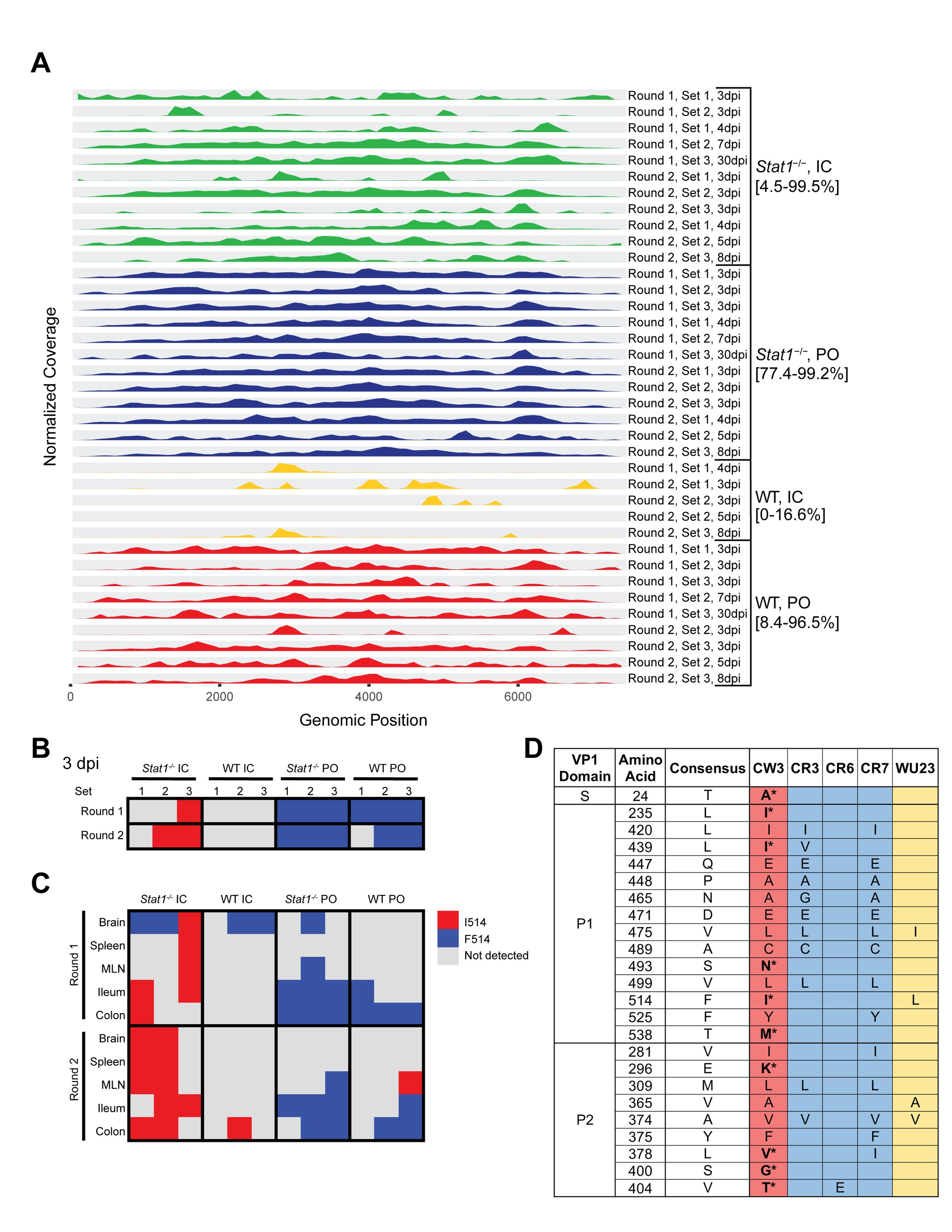

Supplement: S3 Fig — (A) Relative MNoV genomic coverage for those samples for which sequencing libraries could be successfully generated is shown. Range of genome coverage (with a cut-off of 100 reads per base) is shown to the right of each group in brackets. (B, C) Heatmap of Sanger sequencing-based analysis of position 6595 of stool (B) and indicated tissues (C) at 3 dpi as depicted in S1B and S1C Fig, with resulting encoded amino acid depicted by color. (D) Summary of mutations in VP1 found in MNoV CW3 and the most closely-related MNoV strains CR3, CR6, CR7 and WU23. Amino acids are sorted by VP1 domain, with the consensus amino acid (defined as being present at that position in >50% of the MNoV strains listed in Fig 2D) indicated. The amino acid at that position in CW3, CR3, CR6, CR7, and WU23 is listed if it differs from the consensus. Less closely-related MNoV isolates listed in Fig 2D were also aligned, but are not shown here as they do not uniquely possess any deviations from the consensus which are shared by CW3. Bolded text and * indicate variants that are unique to CW3. (TIF) [file ppat.1009402.s003.tif]

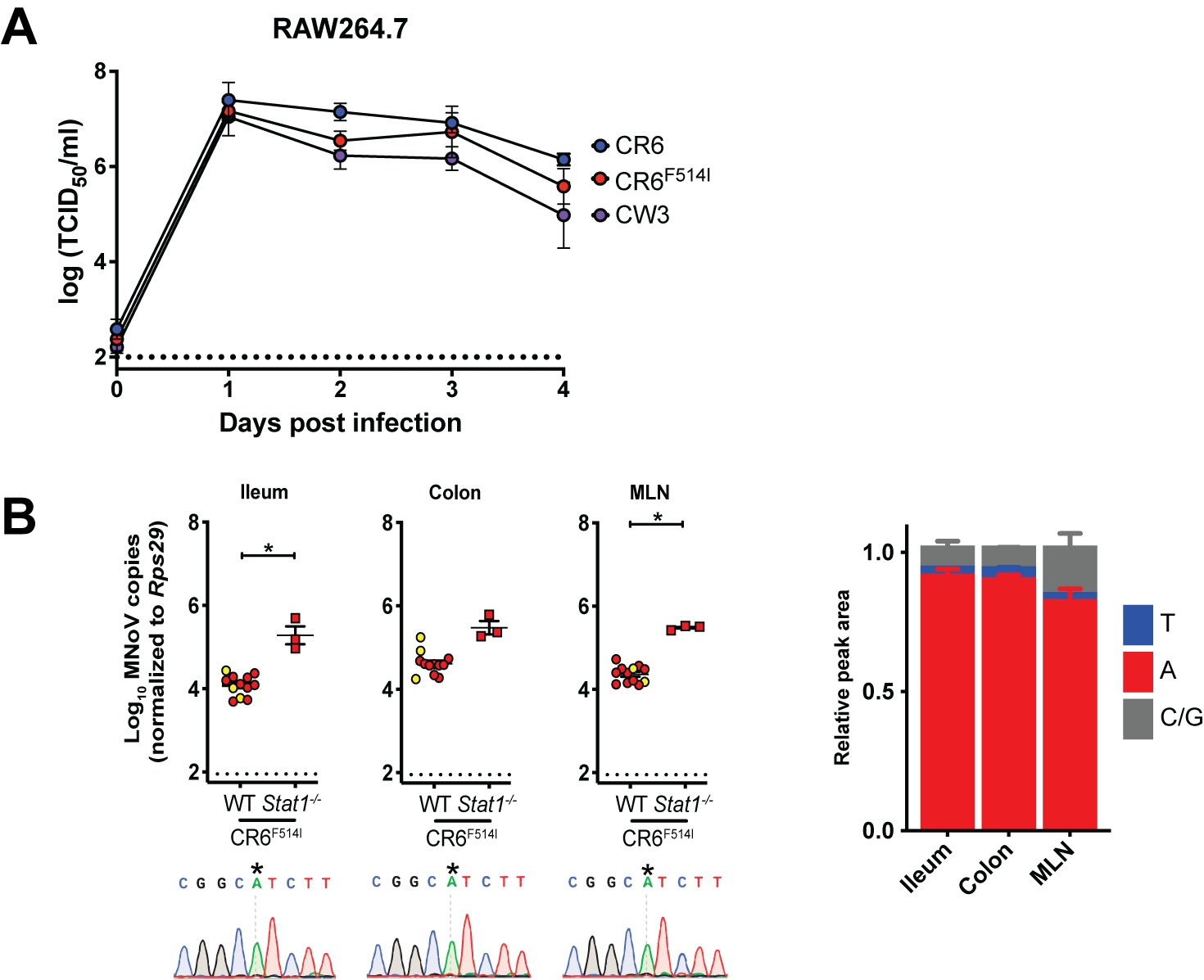

Supplement: S4 Fig — (A) Growth curves of CW3, CR6 and CR6F514I in RAW264.7 cells over 4 days, inoculated at an MOI of 0.05 Results combined from three independent experiments, analyzed by repeated-measures two-way ANOVA. (B) Sanger sequencing analysis of 21 dpi tissues from three independent WT mice infected with CR6F514I, with analyzed tissues depicted in yellow (data repeated from Fig 4B). One representative Sanger sequencing trace is depicted below the graph, with position 6595 indicated with *. Combined data from the three WT mice is shown by averaging the relative peak area for each base at position 6595 from Sanger sequencing traces. (TIF) [file ppat.1009402.s004.tif]

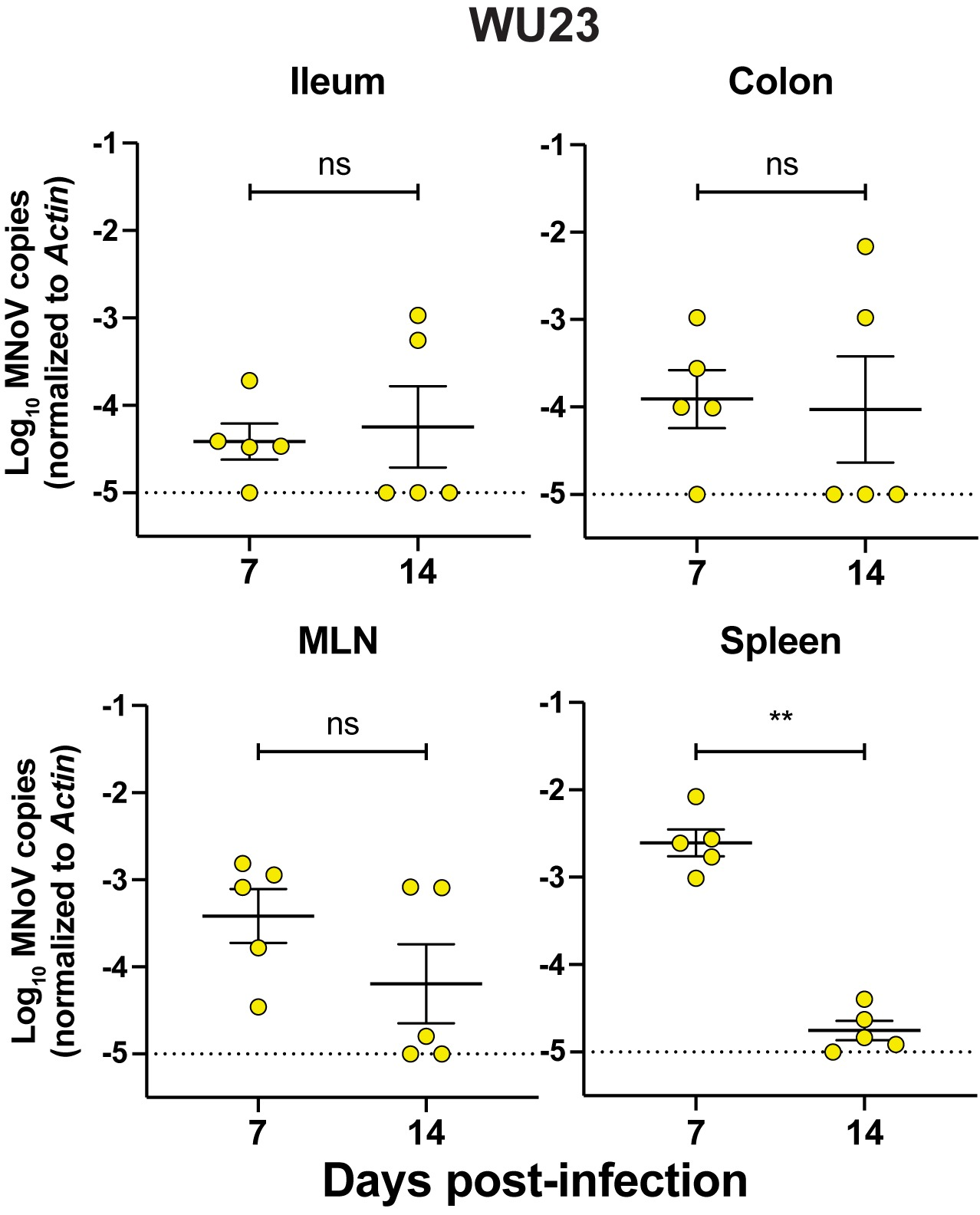

Supplement: S5 Fig — WT mice were orally inoculated with 106 PFU of MNoV WU23. Mice were sacrificed at 7 or 14 dpi, and the indicated organs were harvested and MNoV genome copies were quantified by qPCR. N = 5 mice per group from one experiment, with groups compared by Mann-Whitney test. Data shown as mean +/- SEM. **, P < 0.01; ns, not significant. (TIF) [file ppat.1009402.s005.tif]

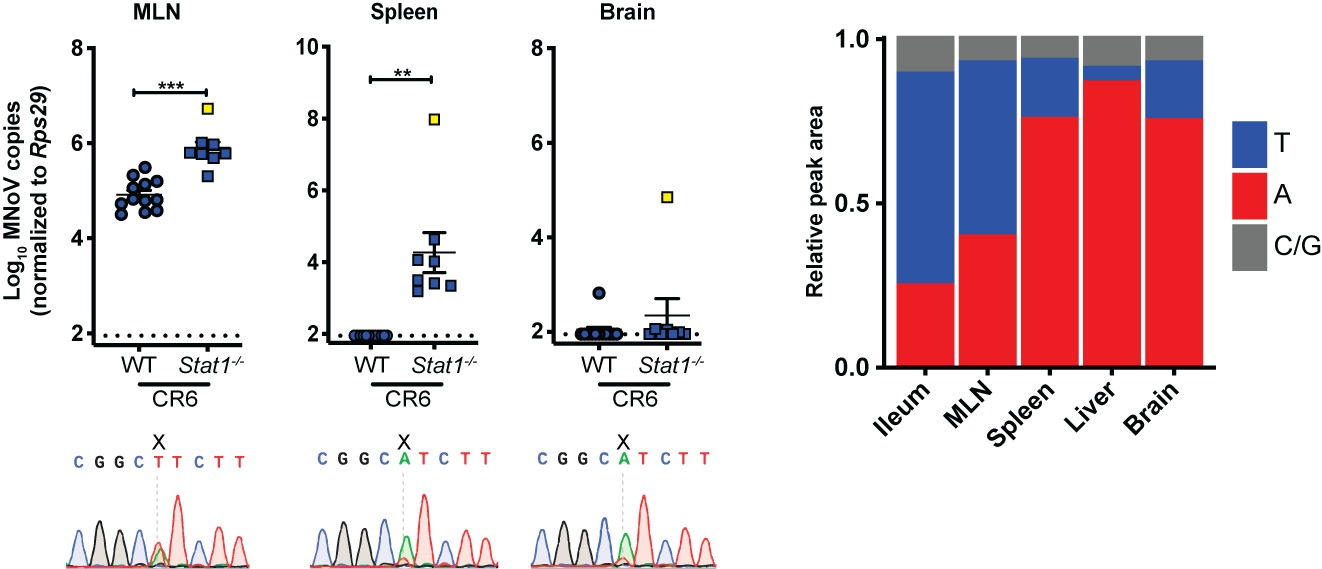

Supplement: S6 Fig — Sanger sequencing analysis of observed 5 dpi single “outlier” from Stat1-/- PO infection with CR6 (data repeated from Fig 3F). qPCR values from the “outlier” mouse are depicted in yellow, and Sanger sequencing traces for the area around nucleotide 6595 from each tissue are depicted beneath the graph for that tissue, with nucleotide 6595 indicated with X. A relative quantification of the abundance of each base from the Sanger sequencing traces is also provided. (TIF) [file ppat.1009402.s006.tif]

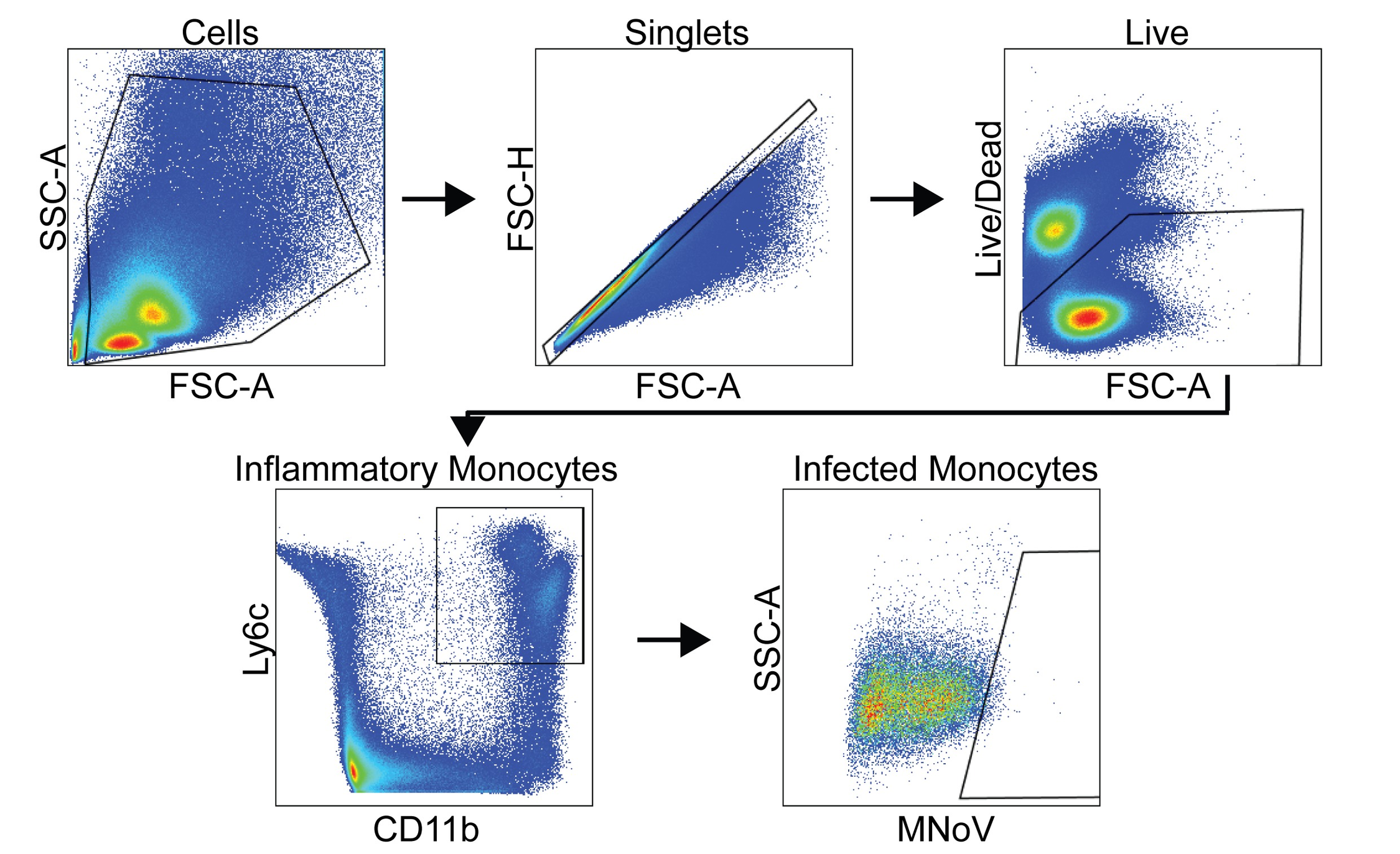

Supplement: S7 Fig — Cells were gated as shown with a representative MLN sample from a Stat1-/- mouse infected with CW3. Cells were gated for live, single cells on the basis of FSC and SSC, followed by live/dead staining with LIVE/DEAD Aqua Stain. Inflammatory monocytes (Fig 7A) were identified as being CD11b+Ly6C+, and infected monocytes (Fig 7B) were identified as those inflammatory monocytes which were positive for MNoV NS1 or NS1/2. (TIF) [file ppat.1009402.s007.tif]

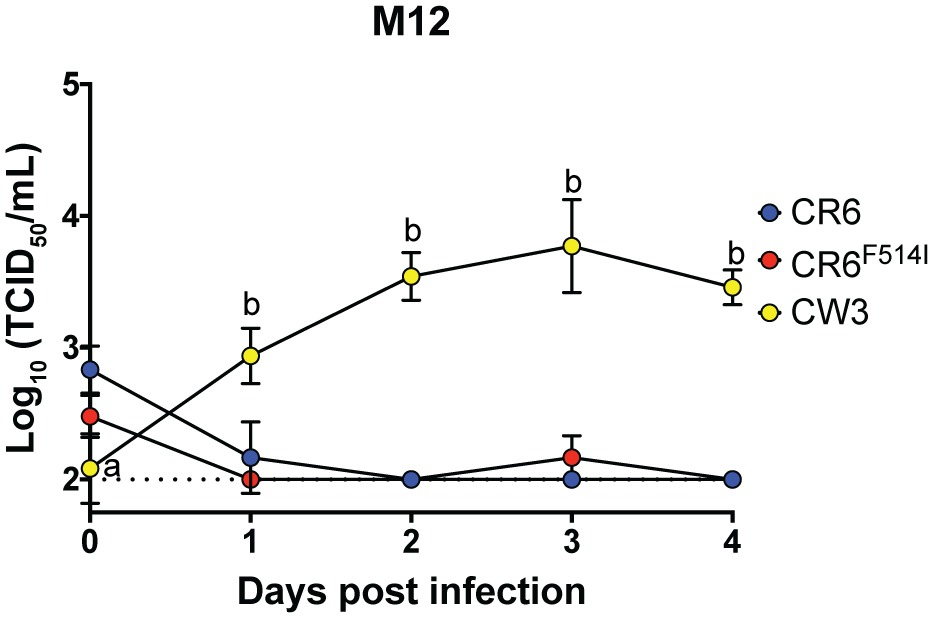

Supplement: S8 Fig — Growth curves of CR6 and CR6F514I in M12 cells over 4 days, inoculated at an MOI of 0.05. Results combined from three independent experiments, analyzed by repeated-measures two-way ANOVA, with Šídák’s multiple comparison test. a indicates significant difference vs. CR6 (P < 0.05); b indicates significant difference vs. CR6 and CR6F514I (P < 0.05). (TIF) [file ppat.1009402.s008.tif]

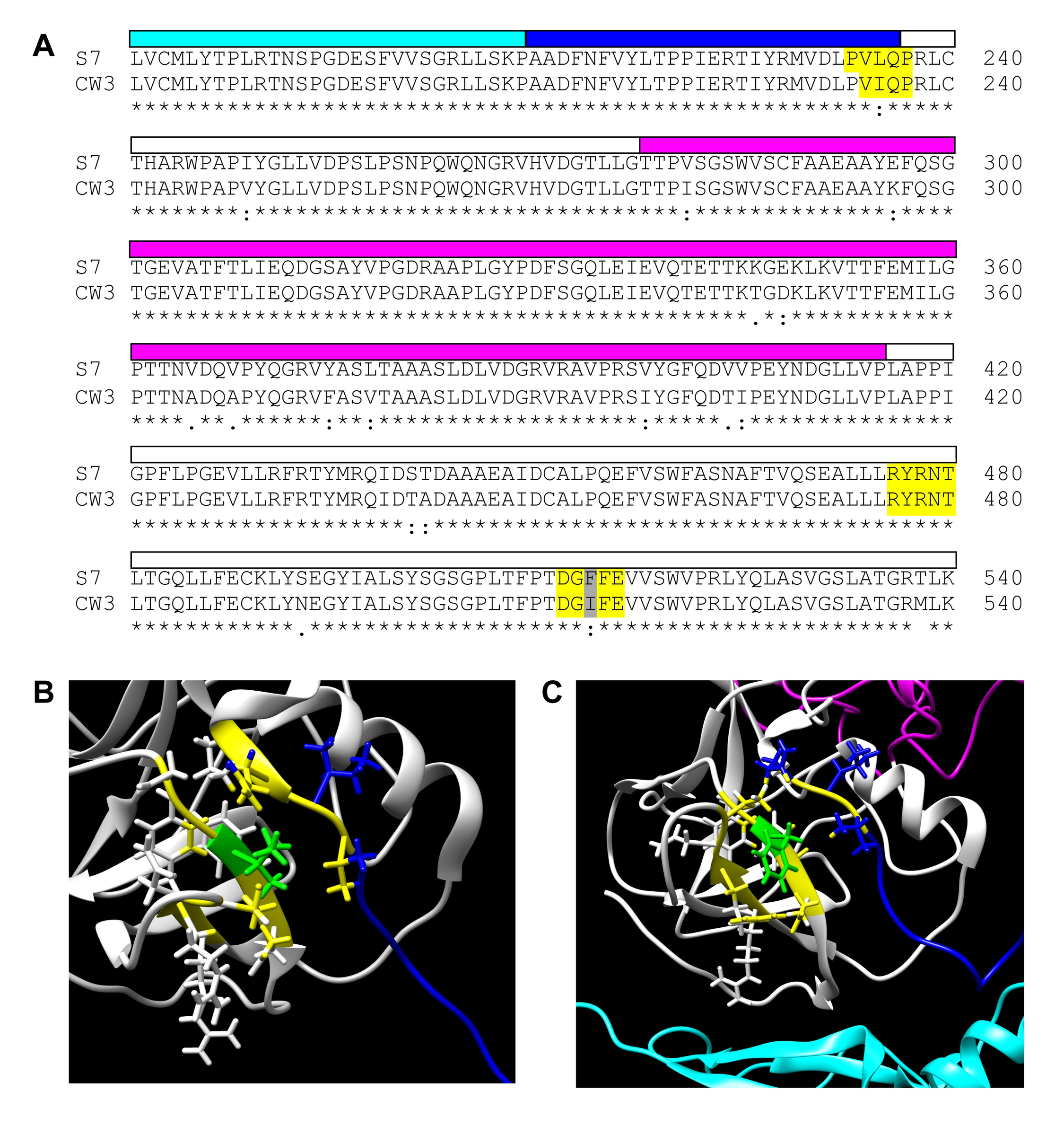

Supplement: S9 Fig — (A) Aligned sequences of MNoV S7 and CW3 VP1 proteins showing sequence conservation and residues with which amino acid 514 may interact. Yellow highlights indicate putative 514-interacting residues (within 4 angstroms, as predicted by UCSF Chimera); grey highlight indicates residue 514. Bars above each row of sequence indicate the domain, colored as in Fig 8, with the shell, P1, and P2 domains in cyan, white, and magenta, respectively, and with the linker region (residues 210–236) additionally shown in dark blue. Alignment performed using Clustal Omega. (B) A view of the structure of MNoV S7 VP1, showing residue F514 (in green) and nearby residues. Domains are colored as in panel A, with residues with which F514 may interact (as listed in (A)) colored in yellow, with side chains additionally shown for those amino acids. (C) A view of the structure of MNoV CW3 VP1, with coloring and visualizations done as for S7 in (B). UCSF Chimera used for all visualizations. (TIF) [file ppat.1009402.s009.tif]

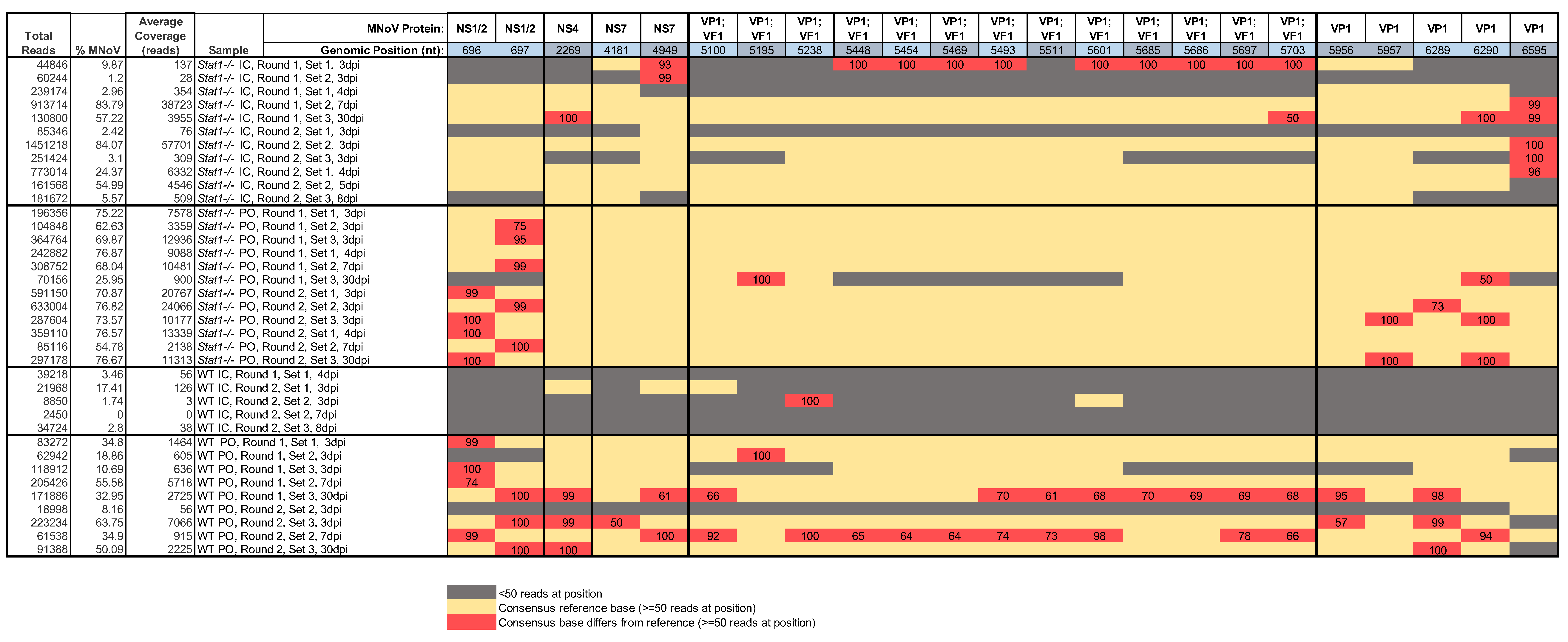

Supplement: S1 Table — Positions throughout the MNoV CR6 genome at which a mutation was observed in more than one mouse are shown. Nucleotides are labeled by position within the genome and the protein(s) within which the mutation is found. Samples are sorted by mouse genotype and round, followed by dpi. Total sequencing reads, percentage of reads identified as MNoV, and average coverage across the MNoV genome are shown for each sample. For each mutation, individual samples are labeled in grey (indicating insufficient coverage at that position to call a mutation at that nucleotide position), yellow (sufficient coverage, and the consensus sequence of that base agrees with the reference), or red (sufficient coverage, with the consensus base differing from the reference, and with the percentage of reads differing from the reference shown in each square). (TIF) [file ppat.1009402.s010.tif]
