# Supplementary material for: Norovirus evolution in immunodeficient mice reveals potentiated pathogenicity via a single nucleotide change in the viral capsid
Source: PLoS Pathog. 2021 Mar 11;17(3):e1009402. doi: 10.1371/journal.ppat.1009402 (PMC7987144; doi:10.1371/journal.ppat.1009402)
Supplement: Attachment — Submitted filename: PLoS_Pathog_Resub_Response_to_Reviewers_FCW_MTB.docx [file ppat.1009402.s011.docx]

**Response to Reviewers for “*Norovirus evolution in immunodeficient mice reveals potentiated pathogenicity via a single nucleotide change in the viral capsid***”

**Editor:**

The reviewers appreciated the attention to an interesting topic and the high quality of the work. A number of modifications aimed at increasing the clarity of the text and figures were suggested, and we encourage you to consider this feedback carefully. In addition, Reviewer 3 raised concerns that the conclusions drawn from Figure 7 are not well supported. Namely, the conclusion that I514 increases systemic virus spread due to increased monocyte recruitment early during infection was questioned. In light of this, we ask that you consider the reviewer's suggested strategies for enhanced analysis of the existing data and follow up with additional experiments if needed.

We are grateful to the editor and reviewers for their helpful comments. We have made a number of suggested edits within the text and figures to improve clarity, described in detail below, and included data about all variants detected in our experimental system. We have carefully scrutinized the data presented in Figure 7 and performed further experiments to fortify our conclusions, and now also provide new complementary Supplemental Figure 8.

**Reviewer #1**

Data were illustrated in numerous figures for each section and because the results are described in the text of each section it might be helpful to move a subset of the figures into a supplementary data section (i.e. Fig 3B and 3E, Fig 4 Sanger traces) to make the paper easier to follow.

We appreciate the reviewer’s comments regarding clarity. We carefully considered whether these data might be better served in a supplemental data section, but because we already have substantial data in the supplement, we ultimately decided to leave the suggested panels within their original figures to minimize flipping between main and supplemental figures for the ease of the reader.

The mouse strain phenotypes used should be briefly described when first mentioned in the manuscript (line 82 and line 353).

We thank the reviewer for pointing this out. Short descriptions of the phenotypes of *Rag2^-/-^Stat1^-/-^* (“which lack both adaptive immunity and innate IFN signaling”) and *Ifnar1^-/-^* mice (“which lack type I IFN signaling”) have been added to the main text. *Pou2f3^-/-^Stat1^-/-^* mice are described in lines 353-355 of the original submission.

Line 180: Why was a 1:80 dilution selected for passage of brain homogenate?

Because we lacked *a priori* knowledge of the infectivity of the brain homogenate, we selected a 1:80 dilution to match our standard dose of 25uL. We have now added this information into the methods: “For brain homogenate infection, a 1:80 dilution of 2-ml brain homogenate was used for volumetric matching to our standard dose of 25 μl.”

Lines 200-209, Suppl. 3A, etc. Data from the deep sequencing is not clearly described. For example, is it known why there is so much variation between samples in sequencing depth across each genome? Were other high frequency variants detected in > 1 sample?

A key factor influencing overall genomic coverage was the concentration of viral genomes in the original samples (Suppl. Fig 2C). Samples with very low viral loads, such as WT IC samples, predictably exhibited very poor coverage, while samples with high viral loads yielded complete or nearly-complete coverage. We have now further clarified this point by adding the statement, “Despite low viral abundance in some samples” to our discussion of the genome coverage yielded, as well as the statement “Although we recovered whole or nearly whole genomes from samples with high viral loads, namely *Stat1^-/-^* mice as well as WT mice infected orally,” (line 209-210) to our discussion of our subsequent Sanger-based confirmation. New **Supplemental Table 1** also provides additional detail about the sequencing reads, percentage of those reads which were MNoV, and the average genomic coverage for each sample.

With regard to variable coverage across the genome for samples with high viral loads, while we had expected to find poor coverage at the ends of this linear genome, which are less likely to be effectively captured and amplified by our Nextera sequencing approach, we do not yet have a definitive explanation for why some regions (e.g. genomic position ~6000-6500) appear to be very well-covered in most samples while others (e.g. genomic position ~5500-6000) consistently exhibited poorer coverage. We speculate that differential coverage could be secondary to variable probe-binding efficiency, probe recruitment by host or bacterial nucleic acid present in the sample, biases in efficiency by the Nextera transposase or PCR steps, or a combination of the above.

The reviewer further raises an important point that other variants were indeed detected in multiple other samples. Our newly-added table **Supplemental Table 1** summarizes all viral variants detected in stool samples from multiple mice. Importantly, none of these other variants exhibited this intriguing pattern of exclusively emerging in the *Stat1^-/-^* IC group.

Line 293: One of 3 mice were analyzed for the emergence of F514I; given the relative ease of Sanger sequencing it might be informative to check additional mice for F514I emergence.

We agree that these data could be interesting, but we concluded that this additional sequencing would not alter our final conclusions regarding emergence and pathogenesis of our novel MNoV variant. We performed this additional sequencing to identify the reason that a lone *Stat1^-/-^* mouse infected with CR6 exhibited such enhanced viral load and morbidity as compared to others in its experiment. Adding additional sequencing would allow us to quantify the percent of mice in which our F514I variant emerges, but demonstrating that it *can* emerge in *Stat1^-/-^* mice infected orally was the primary driver for including these data.

Fig 8 A & B. Without the labeling of the protein domains and the mutation site it is hard to understand what these pictures are illustrating.

We very much agree and apologize for this oversight. We have now added domain labels to the figure itself, in addition to the labels within the figure legend, to clarify this.

A short paragraph in the discussion section on how this MNoV model helps us understand human norovirus disease would increase the general relevance of the finding. For example, do human noroviruses evolve differently in human hosts that are immunocompromised (i.e. due to chronic infections or malnutrition)?

While we have included some information on this topic in the introduction (Lines 79-80), we agree this is an important point. We have now added the statement, “Recent studies have indicated that distinct HNoV variants emerge in immunocompromised hosts, and indeed that this shed virus maintains infectivity [1-4].” to the discussion and included additional relevant references that have explored HNoV variants in immunocompromised hosts (Lines 581-583).

Raw deep sequencing data should be deposited in publicly available database.

The sequencing data has now been deposited to the European Nucleotide Archive, with accession number PRJEB38177 (ERP121566), and a reference to this has been added within the Materials and Methods, Lines 729-730.

**Reviewer #2**

Overall, the work is very exhaustive and performed to a high standard. It conclusively shows the effect of this mutation on pathogenesis in vivo and provides insight into the potential mechanism underlying the effect. Moreover, it shows that a single capsid mutation can have important consequences for pathogenesis, as has been seen in other models (e.g. coxsackievirus B3). I find the fact that this mutation was observed in the receptor knock out mice lacking Stat1 (in a previous Plos Path publication from the authors) very interesting. The connection between an alternative receptor and pathogenesis of this mutation remains to be explored (and its relationship to hematopoietic cells).

We thank the reviewer for their positive comments about our study.

It is not clear to me how easy it would have been to pick up this mutation from sequence alignments, as it appears in figure 2D. It would be helpful to know if multiple mutations are conserved in acute versus persistent infections in P1 or P2. Including such alignment in the supplement would be helpful.

This is a very important point, as if F514I was the only mutation seen uniquely in CW3, it would have been easy to identify it as a residue with an important role in pathogenesis. However, F514I is not the only mutation unique to CW3 among MNoV strains, as there are a total of nine missense mutations that are uniquely seen in CW3. A new panel (**Supplemental Figure 3D**) summarizing additional point mutations seen in CW3, and not in chronic MNoV strains, has been added to clarify this point. We have also added in the accompanying text: “Analysis of the amino acid sequences of VP1 from published MNoV genomes revealed that there was substantial conservation of this protein between persistent and acute MNoV strains, with 88.5% amino acid identity shared between ten characterized isolates. From these sequences, we additionally observed that there were only ten unique amino acid variants in acute, virulent strain MNV-1 and its infectious clone CW3 that were not observed in any of these nonlethal, persistent strains, including the F514I mutation.”

It would be helpful to have a table summarizing mutations that appear at high frequency in the NGS and their frequency assuming sufficient coverage is available. I can’t tell right now how frequent the F514I mutation was in the NGS data (the Sanger suggests this mutation is at 100%), nor the existence of additional mutations that could be of interest. Also, what is the y axis in figure 3A.

We very much agree with the reviewer’s comment. A table (**Supplemental Table 1**) has been added which, in addition to listing additional variants observed in the initial *in vivo* experimental evolution sequencing, also demonstrates the frequency with which mutations were observed in all sequenced stool samples.

Figure S2B/C should indicate how well the curve fits the data, not just the p value (R2).

This was an oversight on our part, and R^2^ values have now been added to this figure.

Line 243 says no differences associated with the mutation, but there is a difference in stat1-/- mice in the MLN (figure 3F).

We thank the reviewer for noting this. We have now corrected this section (now Lines 254-260) to better represent the data, grouping MLNs with other non-intestinal tissues with which it shares similar phenotypes.

It could be interesting to see if the mutant virus is better at infecting hematopoietic cells in vitro than the WT virus from stat1 knock out or WT mice . However, the experiment in figure 7D provides good evidence to suggest this.

This is an important, and very interesting, point. We have now tested whether CR6^F514I^ gains the ability to infect an immortalized B cell line *in vitro* compared to CR6, but found that it did not. We have now added this data as **Supplemental Figure 8**. We speculate that, consistent with our observation that inflammatory monocytes exhibit enhanced infection with CR6^F514I^ in the MLN, that myeloid-derived hematopoietic cells are likely to be the mediators of systemic spread, and agree that further investigations along this line will continue to be important for fully understanding the effects of the F514I mutation.

Minor comments:
1. Missing words in the sentences on lines 87 and 192
2. Define set1 in the text
3. Line 310, white square should be blue square
4. NGS accession codes are missing.

We thank the reviewer for their careful reading of our manuscript. These points have all been corrected within the text, including addition of the NGS accession codes as detailed above, for the reader’s ease.

**Reviewer** **#3**:

Overall, this manuscript is well-written and founded on an interesting scientific premise. The experiments are properly designed and the results are tantalizing. Data showing that the F514I mutation confers a more virulent phenotype similar to acute strains is convincing. The less developed extension of this observation to the L514 polymorphism is less convincing, mainly because this used an isolate and not a recombinant virus with a single polymorphism. The major weakness in the paper is in the authors’ conclusion that the I514 change increases systemic virus spread due to increased monocyte recruitment early during infection, similar to CW3. The authors’ conclusion may ultimately be correct; however, the data presented in Fig 7 are not strong enough to support this conclusion. The point-by-point analysis below may aid in strengthening these data:

Fig 7A,B: The authors quantitate the number of total and MNV-infected monocytes in the MLN and PP at 2 dpi. In Fig 7A, they conclude that CW3 infection exhibits increased monocytes in the MLN and PP, which is consistent with a previous report and supported by statistical analysis. However, they further conclude that CW3 and CR6-F514I exhibited similar levels of monocyte recruitment in the PP. This is based on a non-significant difference between CW3 and CR6-F514I. This is based on using CW3 as the comparator**;** however, it seems more appropriate to use CR6 as the comparator, as it is the polymorphism in CR6 that is being tested. However, the most important comparison is whether CR6-F514I confers increased % monocytes in the PP. That statistical comparison is not provided but is crucial to support their conclusions. There are two problems with this data: The authors show that monocyte levels are high for CW3 than for CR6, but did not do the same analysis for CR6-F514I.

We thank the reviewer for their in-depth comments regarding Figure 7, and have made a number of alterations to this figure in an attempt to improve its quality and clarity. As suggested by the reviewer, CW3 was indeed likely not the best comparator for these experiments. We have now used naïve mice as the comparator, and this has been updated within the figure and its legend (Line 448). Regarding the increased monocyte infiltration into the PPs, after increased scrutiny of our data and additional experimental replicates as described below, we were still unable to demonstrate a statistically significant increase in monocyte counts in these tissues when comparing CR6^F514I^-infected mice to naïve mice. We suggest two reasons for this failure to reach statistical significance. First, there is a high level of variability in this phenotype, as we now discuss further in the text (Lines 417-420): “As reported previously (5), there were high levels of variation between individual mice in these phenotypes, but in sum these data suggest that F514I may partially contribute to the inflammatory monocyte recruitment and infection phenotypes observed with the full CW3 capsid.”

Second, the timing selected for these experiments is likely suboptimal. CW3 is lethal to most IFN-deficient mice by 3 dpi, while CR6^F514I^ takes longer (until about 5 dpi) to achieve significant lethality. This may explain why some CR6^F514I^-infected mice have a more “CR6-like” phenotype in Figure 7A, and some have a more “CW3-like” phenotype, as CR6^F514I^ generally takes ~2 days longer than CW3 to achieve the same virulence. If these data had instead been collected at 4 dpi (which would not be viable for this comparison, since, as stated, most CW3-infected mice would be dead by this timepoint), we would anticipate seeing a much more CW3-like phenotype for our CR6^F514I^-infected mice. While a more careful analysis of the timing of monocyte recruitment and infection proved impractical for our revision experiments, we agree that this will be important for our future studies of F514I.

Additionally, the in Fig 7B, the authors conclude that there is a trend for an increased number of MNV infected monocytes in the MLN of CR6-F514I than in those infected with CR6. However, the is not a clear trend and is strongly driven by a high degree of animal-to-animal variability. Given the authors’ conclusion that I514 increases monocyte recruitment, the experimental evidence must be stronger than a trend.

We agree with the reviewer’s point and have now strengthened our experimental evidence. We repeated these experiments an additional two times, and have found that the MLN data presented in Figure 7B indeed demonstrates a significantly increased % of monocytes infected by CR6^F514I^ or CW3, but not CR6, within the MLNs.

Fig 7C: Another facet of the authors’ conclusion is that the monocyte recruitment is an early event during infection that is driven by a shift in tropism from Tuft cells to monocytes in the Peyer’s Patch. This is tested by measuring the viral genome copies in ileum, colon and Peyer’s Patches on 1 dpi. As for 5 dpi, the results show a clear decrease in genome copies in ileum and colon. However, in the Peyer’s Patch the variability in the data renders the difference non-significant. While this trend is more convincing than that claimed in Fig 7B, the veracity of the conclusion is weakened by this data.

We agree that these data would be more impactful if our statistical testing identified a significant difference in Figure 7C, but in the context of the additional data throughout this figure supporting our conclusions, we suggest that the observed trend is sufficiently clear to add credence to our conclusion.

Overall, in Fig 7A-C, there is considerable variability that confounds clear interpretation of the results. Given the variability between individual animals, it would be reasonable to filter the data with an Outlier statistical analysis. Either Grubbs or ROUT would be acceptable for this. This may require additional animal numbers to maintain power for the statistical analyses.

We agree with the reviewer that there is a high level of variability in these data, as discussed above. We attempted to address this in three ways:

1) by applying more stringent filtering to samples included in these figures. We noted, in carefully reviewing the raw data for this figure, that some samples exhibited low cell counts and/or viability, which may have confounded our results. As such, we removed any samples which had fewer than one thousand total live cells for both MLNs and PP and/or less than 50% cell viability for the MLNs as determined by live/dead staining.

2) by adding an additional two replicates of the described experiments, thereby yielding a net increase of two replicates for monocyte infiltration (Figure 7A) and one replicate for MNoV infection of monocytes (Figure 7B).

3) by removing outliers, as calculated by ROUT, with Q=1%.

With these alterations, we are more confident in the presented data and believe they now more strongly support our conclusions.

While the authors’ work has convinced me that the F514I variant confers a replication advantage in monocytes (and other lymphoid tissue), the bigger questions that are not well address is why and how? As part of the major capsid protein, it is natural to speculate that it involves entry. But then why would entry be more efficient in monocytes than in Tuft cells. Is there any way the nucleotide polymorphism could have its affect at the RNA level rather than the protein level?

The questions proposed by the reviewer are very interesting and will certainly be the subject of future work by our lab. We agree that our lead hypothesis is that this variant affects viral entry, and as noted above, we have now tested whether F514I confers any improved infection of B cells *in vitro* (**Supplemental Figure 8**) and have found that it does not. We are currently exploring whether differential binding of CD300LF or CD300LD may contribute to differential infection of hematopoietic versus tuft cells. Unfortunately, our studies are too preliminary to include here. We also speculate that other factors such as bile acids, which are known to act as cofactors for MNoV-receptor interactions, and which would have different concentrations in lymphoid tissues than in the gut lumen, could contribute to differential tropism of this viral variant. While our mutation does not directly affect known bile acid-binding portions of the protein, some conformational differences may certainly be indirectly conferred. While our studies exploring these interactions, or alternately identifying a post-entry role for our variant as the reviewer suggests, are beyond the scope of the current work, we agree they are critical to explore further.

Is passage in the brain preferential for the F514I variant? Does it also arise quickly after IP or IV inoculation? Does the F514I variant arise even at low levels by in vitro propagation of virus in BV2 cells? Are the IC passage experiments showing a true de novo mutation arising, or is it establishment of a minor variant as the major species due to a considerable replication advantage? Similar, will the F514I variant emerge from any immunodeficient model or is it an exclusive feature of IFN-signaling deficient animals.

We observed development of the F514I variant within a single orally-infected *Stat1^-/-^* mouse, and we have previously observed this mutation in a majority of IP-infected *Stat1^-/-^* mice (Graziano et al., 2020), indicating that it is not unique to IC infections. Its relative frequency of arising in IC versus IP infections is not known, but it seems to arise commonly in both contexts.

Regarding whether this variant is observed *in vitro* and whether it’s truly *de novo*: This variant was not observed within our TC-derived inoculum or at low frequency in WT mice within our initial passaging and sequencing studies. It may be present at very low percentages within all of our mice, but if so, it was generally below our limit of detection and IFN-mediated regulation is sufficient to prevent it from becoming a dominant variant. As such, we speculate that it is indeed a *de novo* mutation, but cannot absolutely confirm this.

Regarding emergence of this variant in other lines, we have solely seen it arising within *Stat1^-/-^* mice. However, we do find the question of whether it may emerge in additional immunodeficient mice very intriguing and are enthusiastic to attempt similar experiments in additional lines. In addition to determining which aspect of IFN signaling (type I, II, or III) is key for limiting this variant, we also plan to assess viral evolution in lines such as muMT or *Cd8^-/-^* mice which lack additional key aspects of the immune system.

Since F514I seems to arise frequently. Does having this variant as part of the quasispecies benefit parental CR6 in some way?

Since we almost exclusively observe this variant emerging in the absence of IFN signaling, we speculate that it is not routinely part of the quasispecies in WT mice, and instead its emergence secondary to *de novo* mutation normally kept in check by IFNs. However, the observation that CR6^F514I^ is able to be persistently maintained in the WT mouse intestine suggests it is not wholly detrimental. Though out-of-scope for our current study, we agree this is an intriguing question to pursue and will plan to initiate co-infection experiments between CR6 and CR6^F514I^ as part of our continuing explorations of the effects of this amino acid change.

The data in Fig 5 are interesting and clearly show a replication detriment in CW3 with an I514F mutation. However, is it surprising that CW3 doesn’t replicate more like CR6?

We agree that while CW3-I514F clearly shows a defect in extraintestinal/brain spread compared to CW3 in IFN-signaling deficient mice, it fails to replicate some of the CR6 phenotypes observed in WT mice. Some of this may be due to the different experimental designs between the experiments shown in Figures 3 and 5, as mice in figure 3 (infected with CR6 or CR6^F514I^) were sacrificed at 5 dpi, while mice in figure 5 (infected with CW3 or CW3^I514F^) had to be sacrificed earlier, at 3 dpi, due to extensive weight loss in CW3-infected animals. However, we do still see increased colonic levels of CW3-I514F, partially phenocopying CR6. Importantly, while here we have focused on viral capsid differences, other studies have convincingly demonstrated that CW3 and CR6 differences in WT mice are mediated by both VP1 and NS1/2 changes, and indeed that secreted NS1 is required to promote persistence by suppressing IFN-lambda signaling [5, 6]. Our interpretation is that because we did not also replace the CW3-I514F with a CR6 NS1/2, we are not able to observe robust colonic replication with this viral strain. To address this point, we have added the sentence, “This NS1 requirement for persistence and robust colonic replication also likely underlies the lack of differential colonic replication observed in WT mice infected with CW3^I514F^ compared to CW3.” to the discussion.

In the Tuft cells studies (Fig 6), the mice infected with CR6-F514I exhibited ~50% survival in both the Pou2f3+/- and Pou2f3-/- mice on the STAT1-/- background; however, in the previous Fig there was only ~15-20% survival in STAT1-/- mice. This seems like a large difference given a largely similar genetic background. What is driving this difference?

We certainly agree with the reviewer’s excellent observation that there is differential lethality between experiments. Importantly, lethality experiments conducted in *Stat1^-/-^* mice in Fig. 1 and 3 were performed at Washington University, while studies analyzing lethality in *Pou2f3^+/-^Stat1^-/-^* and *Pou2f3^-/-^Stat1^-/-^* mice in Fig. 6 were conducted at Yale University. At this stage, we can only propose that environmental factors such as diet, microbiota, or the presence/absence of endemic viruses such as astrovirus, could have contributed to these facility-mediated differences, perhaps permitting a subset of mice to survive long enough for adaptive immunity to help limit lethality. While we do not know the specific cause, we have now further specified that experiments with these lines were “conducted at Yale University” in our methods section.

Fig 1C: Consider a rainbow heatmap. The single color, particularly red, is very difficult to visualize the small gradations.

We appreciate the reviewer’s suggestion and have updated the heatmaps within **Figure 1** and **Supplemental Figure 1** to a blue-to-red heatmap to make these differences easier to visualize.

Lines 87-89: Could also contrast the limited genotype diversity among MNV with the high diversity among HuNoV.

Lines 127-128: The last sentence should be revised to be more precise. What elements of pathogenesis have you revealed?

Lines 239-240: This sentence reads awkwardly to me. Perhaps: “When administered to Stat-/- mice PO, CR6-F514I induced significant weight loss, as well as greater splenomegaly and lethality than CR6”

**Note, in Fig 3C there is no statistical analysis of spleen weight between CR6 and CR6-F514I. This needs to be added because the comparison is written into the text in lines 239-240.

Lines 274-275: read awkward. Perhaps: “only a limited cohort. Surviving mice regained some weight but remained underweight compared to other cohorts.”

We thank the reviewer for these comments, and we have now incorporated these edits throughout to improve our manuscript.

Text on Lines 87-89 now reads: “Although there is substantial genetic diversity between HNoV isolates, with human-infecting strains found within three of seven NoV genogroups, there is limited diversity among the numerous reported MNoV strains, all of which are within a single genogroup [7]. In spite of the limited (<13%) nucleotide divergence between MNoV strains, there are large strain-specific phenotypic differences [6, 8, 9].”

Text on Lines 130-133 now reads: “Our approach revealed a critical role for the capsid in determining key aspects of viral pathogenesis, including cell and tissue tropism and lethality, outside of the well-appreciated importance of the receptor-binding domain in these phenotypes.”

Lines 239-240 adjusted as suggested, with statistical analysis now adjusted.

Line 284-286 now reads: “Few *Stat1^-/-^* mice infected with CR6^F514I^ survived past 7 dpi, permitting analysis of only a limited cohort; those that survived regained some weight, but remained underweight compared to other cohorts (Figure 4A).”

**References for Response**

1.	Hasing ME, Hazes B, Lee BE, Preiksaitis JK, Pang XL. A next generation sequencing-based method to study the intra-host genetic diversity of norovirus in patients with acute and chronic infection. BMC Genomics. 2016;17:480. Epub 2016/07/02. doi: 10.1186/s12864-016-2831-y. PubMed PMID: 27363999; PubMed Central PMCID: PMCPMC4929757.

2.	van Beek J, de Graaf M, Smits S, Schapendonk CME, Verjans G, Vennema H, et al. Whole-Genome Next-Generation Sequencing to Study Within-Host Evolution of Norovirus (NoV) Among Immunocompromised Patients With Chronic NoV Infection. J Infect Dis. 2017;216(12):1513-24. Epub 2017/10/14. doi: 10.1093/infdis/jix520. PubMed PMID: 29029115.

3.	Vega E, Donaldson E, Huynh J, Barclay L, Lopman B, Baric R, et al. RNA populations in immunocompromised patients as reservoirs for novel norovirus variants. J Virol. 2014;88(24):14184-96. Epub 2014/10/03. doi: 10.1128/JVI.02494-14. PubMed PMID: 25275120; PubMed Central PMCID: PMCPMC4249157.

4.	Davis A, Cortez V, Grodzki M, Dallas R, Ferrolino J, Freiden P, et al. Infectious Norovirus Is Chronically Shed by Immunocompromised Pediatric Hosts. Viruses. 2020;12(6). Epub 2020/06/11. doi: 10.3390/v12060619. PubMed PMID: 32516960; PubMed Central PMCID: PMCPMC7354526.

5.	Lee S, Liu H, Wilen CB, Sychev ZE, Desai C, Hykes BL, Jr., et al. A Secreted Viral Nonstructural Protein Determines Intestinal Norovirus Pathogenesis. Cell Host Microbe. 2019;25(6):845-57 e5. Epub 2019/05/28. doi: 10.1016/j.chom.2019.04.005. PubMed PMID: 31130511; PubMed Central PMCID: PMCPMC6622463.

6.	Nice TJ, Strong DW, McCune BT, Pohl CS, Virgin HW. A single-amino-acid change in murine norovirus NS1/2 is sufficient for colonic tropism and persistence. J Virol. 2013;87(1):327-34. doi: 10.1128/JVI.01864-12. PubMed PMID: 23077309; PubMed Central PMCID: PMCPMC3536416.

7.	Vinje J. Advances in laboratory methods for detection and typing of norovirus. J Clin Microbiol. 2015;53(2):373-81. doi: 10.1128/JCM.01535-14. PubMed PMID: 24989606; PubMed Central PMCID: PMCPMC4298492.

8.	Thackray LB, Wobus CE, Chachu KA, Liu B, Alegre ER, Henderson KS, et al. Murine noroviruses comprising a single genogroup exhibit biological diversity despite limited sequence divergence. J Virol. 2007;81(19):10460-73. Epub 2007/07/27. doi: 10.1128/JVI.00783-07. PubMed PMID: 17652401; PubMed Central PMCID: PMCPMC2045448.

9.	Strong DW, Thackray LB, Smith TJ, Virgin HW. Protruding domain of capsid protein is necessary and sufficient to determine murine norovirus replication and pathogenesis in vivo. J Virol. 2012;86(6):2950-8. doi: 10.1128/JVI.07038-11. PubMed PMID: 22258242; PubMed Central PMCID: PMCPMC3302348.
